# The Extreme Variety of Genotoxic Response to Benzo[a]pyrene in Three Different Human Cell Lines from Three Different Organs

**DOI:** 10.1371/journal.pone.0078356

**Published:** 2013-11-08

**Authors:** Camille Genies, Anne Maître, Emmanuel Lefèbvre, Amandine Jullien, Marianne Chopard-Lallier, Thierry Douki

**Affiliations:** 1 Laboratoire « Lésions des Acides Nucléiques », Université Joseph Fourier – Grenoble 1/CEA/Institut Nanoscience et Cryogénie/SCIB, Grenoble, France; 2 Equipe EPSP Environnement et Prédiction de la Santé des Populations – laboratoire TIMC (UMR CNRS 5525), CHU de Grenoble, Université Joseph Fourier, UFR de médecine, Domaine de la Merci, La Tronche, France; 3 Agence de l′environnement et de la Maîtrise de l′Energie 20, Angers, France; University of Pittsburgh, United States of America

## Abstract

Polycyclic aromatic hydrocarbons (PAHs) are associated with occupational exposure and urban atmospheric pollution. Determination of the genotoxic properties of these compounds is thus of outmost importance. For this purpose a variety of cellular models have been widely used. Reliable results can however only be obtained with models reflecting the specific sensitivity of different organs towards PAHs. In this work, we compared the response to benzo[a]pyrene in cell lines from human lungs (A549) and bladder (T24); two important target organs for PAHs-induced cancer. Human hepatocytes (HepG2) were used as a reference, although liver is not a concern for PAHs carcinogenesis. Adducts arising from the ultimate diol-epoxide metabolite of B[a]P, BPDE, were found to be produced in a dose-dependent manner in HepG2. BPDE DNA adducts were not detected in T24 and in A549 their formation was found to be most efficient at the lowest concentration studied (0.2 µM). These results are probably explained by differences in induction and activity of phase I metabolization enzymes, as well as by proteins eliminating the B[a]P epoxide in A549. In addition to BPDE adducts, oxidative DNA damage, namely strand breaks and oxidized purines were measured and found to be produced only in minute amounts in all three cell lines. In summary, our results emphasize the large differences in the response of cells originating from different organs. Our data also point out the importance of carefully selecting the doses used in *in vitro* toxicological experiments. The example of A549 shows that working at high doses may lead to an underestimation of the risk. Finally, the choice of method for evaluating genotoxicity appears to be of crucial importance. The comet assay does not seem to be the best method for a compound like B[a]P which induces stable adducts causing limited oxidative damage.

## Introduction

Atmospheric pollution concerns numerous occupations but also the general population and includes a wide variety of chemical substances. Among these, polycyclic aromatic hydrocarbons (PAHs) arise mainly from vehicle exhaust, cigarette smoke, residential heating and industry by incomplete combustion of organic matter or in processes using charcoal or petroleum derivatives [1], [2]. Some PAHs are suspected or known human carcinogens and exposure to these compounds is associated with increased cancer incidence, especially in case of occupational exposure [3]. Assessing the deleterious properties of PAHs is thus a major issue in public health. Due to the diversity of the chemical structures of PAHs with more than 100 compounds identified this is however not a straightforward task. In addition, the different PAHs are not all equally carcinogenic and basic toxicological data cannot be extrapolated from one to the other. Another specificity of PAHs is that they are always emitted in complex mixtures whose composition depends on the source. Although a risk assessment strategy based on Toxic Equivalent Factors is applied [4], it does not take into consideration interactions between different PAHs and thus needs to be refined. Consequently, numerous pieces of information concerning the genotoxicity of PAHs remain to be gathered. Animal studies appear to be powerful tools for this purpose. Such studies are however cumbersome and expensive, and regulations tend to limit their use. *In vitro* approaches are thus attractive alternatives [5]. Such investigations may be informative but should take into consideration the target organs of the studied compounds.

In spite of an extensive literature on the toxicity of PAHs, only limited work has been devoted to a systematic comparison between the responses of different human cell lines. This is yet a key issue in modern toxicology. So far, the best documented target organ of PAHs-induced cancer is the lungs [6] although evidence has also been obtained for a role of PAHs in the induction of skin [7] and bladder cancer [8]. We designed the present study to determine whether PAHs, and in particular benzo[a]pyrene (B[a]P), were equally genotoxic in cell lines originating from lungs and bladder, with hepatocytes as a reference metabolizing model. B[a]P is the only PAH classified in group 1 by the International Agency for Research on Cancer (IARC) and considered as a known carcinogen to humans [9]. B[a]P has thus been extensively studied and constitutes the reference compound for assessing toxicity of exposure to mixtures in the Toxic Equivalent Factors approach [4]. The carcinogenic properties of PAHs, and B[a]P in particular are mainly explained by their ability to induce DNA damage. Two main types of DNA lesions have been described, both involving the cellular metabolism aimed at eliminating PAHs [10]. First, the oxidative stress associated with the activity of some metabolization enzymes or by the redox properties of some metabolites can induce DNA strand breaks and oxidized bases. Second, reactive compounds produced during the phase I metabolism, are expected to be conjugated with hydrosoluble groups by transferases, and may add to DNA bases yielding covalent adducts.

Metabolic detoxification has been extensively studied in the case of B[a]P. Cytochrome P450-dependent oxygenases (CYP450), especially 1A1 and 1B1 isoforms, metabolize B[a]P into epoxides. These reactive compounds can be detoxified as glutathione conjugates by glutathione-*S*-transferases (GST). Epoxides are also converted into dihydrodiol derivatives by epoxide hydrolase (EH) and further oxidized by CYP450 to produce B[a]P diol epoxide (BPDE). Most of this ultimate carcinogenic metabolite is detoxified by GST and other phase II enzymes, but it may react in part with DNA. Several types of adducts are thus produced; the predominant one (BPDE-*N^2^*-dGuo) results from addition of BPDE to the N2 exocyclic amino group of guanine [11], [12]. Evidence has also been provided for the formation of oxidative DNA lesions as the result of the production of reactive oxygen species upon metabolization of B[a]P [13], [14], [15]. However, we recently showed that BPDE-*N^2^*-dGuo adducts were produced in an almost two orders of magnitude larger frequency than oxidative strand breaks in human hepatocytes [16].

Obviously, these observations greatly depend on the metabolic properties of the considered organs and cell types. A large number of *in vitro* studies have been carried out using hepatocyte cell lines such as human HepG2, mainly because they possess a large number of enzymatic activities involved in the bioactivation of xenobiotics and of PAHs in particular [17], [18]. Since the liver is not a target organ of PAHs, studies aimed at predicting genotoxic effects should however involve cells more relevant to human health issues. In the present work, we investigated the cytotoxicity and genotoxicity of B[a]P in A549 alveolar epithelial lung cells and in T24 bladder cells which are human cell lines representative of two target tissues. The results were compared to those obtained with HepG2 hepatocytes. The link between the formation of DNA damage and the metabolizing potential of the different cell lines was studied. For this purpose, a wide variety of biological endpoints were monitored, including formation of BPDE-*N^2^*-dGuo, induction of oxidative DNA damage, modulation of the expression of genes involved in metabolization and variation in EROD and GST activities.

## Materials and Methods

### 1. Products and Chemicals

Cell culture reagents including culture media, fetal bovine serum, MEM- non essential amino acids, L- glutamine, PBS, trypsin-EDTA, trypsin 10X, collagen type I rat tail and antibiotics were purchased from Life Technologies (Carlsbad, CA). Dimethylsulfoxide (DMSO), deferoxamine, RNase A, RNase T1, phosphodiesterase I, DNase II, phosphodiesterase II, nuclease P1, alkaline phosphatase, agarose low melt, 1-chloro-2,4-dinitrobenzene (CDNB), cell lytic M Cell lysis agent, ethoxyresorufin and resorufin were obtained from Sigma Aldrich (St Quentin-Fallavier, France). MESA Blue qPCR Mastermix for SYBR Assay Low ROX was provided by Eurogentec (Angers, France). B[a]P (Sigma) was suspended in DMSO and the stock solution was kept at −20°C and diluted directly in culture medium when needed. Solvents used for HPLC- mass spectrometry were of analytical grade.

### 2. Cell Culture

#### 2.1 Cell lines and culture conditions

HepG2 human hepatocyte cell line was supplied by ATCC (HB-8065). A549 human alveolar cells and T24 urinary bladder cell line were purchased from CLS Cell Lines Services, Germany. HepG2 cells were grown in Dubelco's Modified Eagle Medium with a preliminary collagen type I coating of the flasks. A549 cells were grown in a DMEM F12 medium while T24 cells were grown in a Mc Coy's 5A Medium. All media were supplemented with 10% of fetal calf serum, 1% of non-essential amino acids, 1% of L-Glutamine and antibiotics (penicillin 0.01 U/mL, streptomycin 0.01 µg/mL).

#### 2.2. Treatment

Cells were seeded at a density of 27 000 cells/cm^2^ and incubated for 48 h (37°C, 5% CO_2_). Semi-confluency cells were exposed to B[a]P solutions prepared in culture medium without serum. A concentrated solution of B[a]P in DMSO was used for this purpose. Cells exposed to treatment medium with 0.5% of DMSO were used as negative controls. Measurements were performed after 14 h of incubation for dose effect assessment as described by Tarantini *et al* [16] with 0.2, 2 and 5 µM of B[a]P. Several time points ranging between 30 min and 24 h with 0.2 µM of B[a]P were chosen for study of the time-dependence of the cellular response. Finally, after incubation, cells were harvested using trypsine-EDTA and stored as dry pellets at -80°C until analysis for adducts quantification, RT-qPCR analysis or enzymatic activities measurement.

### 3. BPDE-*N^2^*-dGuo quantification

#### 3.1 DNA extraction and hydrolysis

Buffer A (sucrose 320 mM, MgCl_2_ 5 mM, Tris-HCl 10 mM, deferoxamine 0.1 mM and Triton 1%) was added to the cellular pellet. After lysis of the plasmid membranes lysis by use of a vortex, the nuclei were collected after centrifugation (1500×g, 5 min). Nuclear membrane lysis was achieved by buffer B (EDTA-Na_2_ 5 mM, Tris HCl 5 mM, deferoxamine 0.15 mM, SDS 10%) and RNA was removed by incubation with a RNAse mixture (RNAse A 100 mg/mL and T1 1 U/µL) for 15 min at 50°C. Then, proteins were hydrolyzed by incubation for 1 h at 37°C after addition of protease (20 mg/mL). DNA was precipitated by addition of 2× volumes of a NaI solution (7.6 M NaI, 40 mM Tris, 20 mM EDTA-Na_2_, 0.3 mM defferoxamine pH 8) and 3.3× volumes of absolute isopropanol. The sample was washed first with 40% isopropanol then with 70% ethanol and finally resuspended in 50 µl of an aqueous deferoxamine mesylate solution (0.1 mM). Subsequently, the DNA hydrolysis was performed in two successive steps. First, a cocktail of enzymes containing phosphodiesterase II (0.05 U), DNAse II (0.5 U) and nuclease P1 (0.5 U) together with buffer (200 mM succinic acid, 100 mM CaCl_2_ pH 6) was added to the sample. After 2 h incubation at 37°C, the pH was raised from 6 to 8 by addition of buffer (500 mM Tris-HCl, 1 mM EDTA pH 8). The sample was then incubated further for 2 h at 37°C following addition of phosphodiesterase I (0.05 U), alkaline phosphatase (2 U). The reaction was stopped with HCl 0.1 N. After centrifugation (5000×g, 5 min), the DNA hydrolysate was collected, lyophilized and resuspended in MeOH/H_2_O (50/50 v/v) for HPLC-tandem mass spectrometry (HPLC-MS/MS) analysis.

#### 3.2 HPLC-MS/MS

A high performance liquid chromatography-tandem mass spectrometry system (HPLC-MS/MS) was used to quantify BPDE-*N^2^*-dGuo adducts as previously described [19]. The HPLC separation was performed on a 150×2 mm ID C18 reverse phase Uptisphere ODB column (Interchim, Montluçon, France). A gradient of acetonitrile from 0 to 100% in a 2 mM ammonium formate aqueous solution was used. Under these conditions, the retention time of the BPDE-*N* ^2^-dGuo adduct was 18 min. Quantification was performed with a tandem mass spectrometer (API 3000, SCIEX) operated in the positive electrospray ionization mode. Detection was performed in the multiple reaction monitoring mode using specific fragmentations: m/z 570→454, 570→285 and 570→257. In addition to that of adducts, the amount of unmodified nucleosides was determined by using a UV detector set at 260 or 300 nm placed upstream of the inlet of the mass spectrometer. For both types of analytes, quantitative results were obtained by external calibration based on the injection of known amounts of standards. Calibration of the response of the detector was performed for each run of analyses. Repeated injections of standards during the run permitted to control the stability of the sensitivity of the detection and of the retention times.

### 4. Comet assay

Single- and double-strand breaks together with alkali-labile sites were quantified using single-cell alkaline gel electrophoresis technique as previously described [16]. Suspensions of 1500 cells in low melt agarose (0.6%) were deposited on microscopic slides in triplicate for each condition and covered with 1% of agarose. Cell lysis was achieved in a buffer (2.5 M NaCl, 0.5 M Na_2_-EDTA, 10 mM Tris, 1% Sodium lauryl sulfate, 1% Triton X-100 and 10% DMSO, pH 10) for 1 h at 4°C in the dark. Slides were washed 3 times with a reaction buffer (40 mM HEPES, 0.1 M KCl, 0.5 mM EDTA and 200 mg BSA). After the washing step, slides were immersed 30 min in an alkaline electrolysis buffer (300 mM NaOH, 1 mM Na_2_-EDTA, pH 13) for DNA unwinding. Electrophoresis was performed at 25 V/300 mA for 30 min migration. After 3 rinses with neutralizing buffer (0.4 M Tris-HCl, pH 4), DNA was stained with ethidium bromide (20 µg/mL). Some samples were also incubated with Formamidopyrimidine-DNA-*N*-glycosylase (Fpg) to reveal the presence of oxidized purines. In this version of the Comet assay, slides were incubated after lysis for 45 min at 37°C in Fpg buffer (0.1 M KCl, 0.5 mM EDTA-Na_2_, and 0.04 M Tris-HCl, pH 8) in the presence of 0.17 µg of Fpg. Slides were then treated as described above for the standard Comet protocol. For both versions of the assay, slides were analyzed with a fluorescence microscope coupled with a computer system using Comet Assay IV software. We randomly picked 50 comets on each slide and the percentage of tail intensity (% tail intensity) was determined. Results were expressed as an average of scores arising from three different slides for each sample. For calibration purposes, cells exposed to 2 Gy of γ-radiation (2 Gy/min emitted by a ^60^Co source) were analyzed. The increase in % tail intensity was defined as corresponding to 2000 breaks per cell, knowing that 1 Gy is expected to induce 1000 DNA strand breaks in mammalian cells [20], [21], [22]. This correlation was used to calculate the extent of strand breaks and Fpg-sensitive sites in B[a]P treated cells.

### 5. Gene induction of metabolization enzyme

Gene expression was quantified by RT-qPCR using a protocol previously described [23]. Total RNA was extracted from cell pellets using the GenElute mammalian total RNA miniprep kit (Sigma) following the manufacturer's protocol with optional DNAse treatment step. Reverse transcription of RNA in cDNA was performed on 2 µg of RNA samples (Superscript II Reverse transcriptase) and 20 ng were amplified by PCR using gene specific primers (CYP1A1, 1A2, 3A4, 1B1, EH, GST A1 and P1, AKRC1 and MRP4). Sequences of the primers are presented in supplementary data (Table S1). Quantitative PCR was performed in a MX3005P multiplex quantitative PCR system (Stratagene) using MESA Blue qPCR Mastermix for SYBR Assay Low ROX. S18 and GAPDH were chosen as housekeeping genes for normalization and amplified in triplicate for each assay. Variability of genes expression among the various conditions was assessed by Bestkeeper, an Excel based pair-wise RNAm correlation tool with cycle threshold (Ct). RNA level modulation of target genes was computed using the Relative expression Software tool based on Ct comparison.
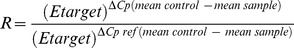



### 6. GST activity

After harvest, cells were lysed using cell lytic M Cell lysis agent mixed with a protease inhibitor cocktail. Samples were then centrifuged at 4°C for 10 min at 2000×g and for 20 min at 16000×g. Activity was measured in the supernatant. Cytosolic GST activity was assessed according to the method of Habig *et al* [24]. CDNB was used as a substrate in the presence of GSH. The reaction medium contained 50 mM potassium phosphate buffer (pH 7.4), 1 mM EDTA, 6 mM GSH and 1 mM CDNB. After addition of 25 µL of diluted cellular extract (1∶4 or 1∶10 v/v), the formation of a GSH-CDNB complex, which is catalyzed by the GST activity present in the sample, was monitored for 5 min by spectroscopic measurement at a 340 nm wavelength. The protein content of the supernatant was measured by the BCA assay. Activity was expressed in µmol of product/min/mg of proteins.

### 7. EROD activity

A549 and HepG2 cells were seeded at 50 000 cells per well in black 96 well plates and incubated for 24 h in culture medium. They were then exposed for 24 h (HepG2) or 48 h (A549) to increasing B[a]P concentrations in DMEM treatment medium. Medium was then removed and replaced by 7-ethoxyresorufin-containing medium (2 µM in culture medium without serum) for 15 min. Resorufin production was then measured by fluorescence on a microplate spectrofluorimeter (Spectra Max M2, Molecular Devices) with excitation and emission wavelengths set at 535 and 590 nm, respectively. Plates were washed twice with PBS and frozen for 45 min at −80°C. Protein content was determined by fluorescamine assay as described by Lorenzen *et al*.[25] EROD activity was expressed in picomoles of resorufin per minute and per milligram of protein (pmol/min/mg protein).

### 8. Statistical analysis

All experiments were performed three times and analyses were each time performed in triplicate. Statistical analysis was conducted using the Student test for qPCR studies. Mann-Whitney tests were applied for all other experiments. Differences were considered as significant when p values were below 0.05.

## Results

### 1. Formation BPDE-*N^2^*-dGuo adducts in HepG2, A549 and T24

**Figure 1:**
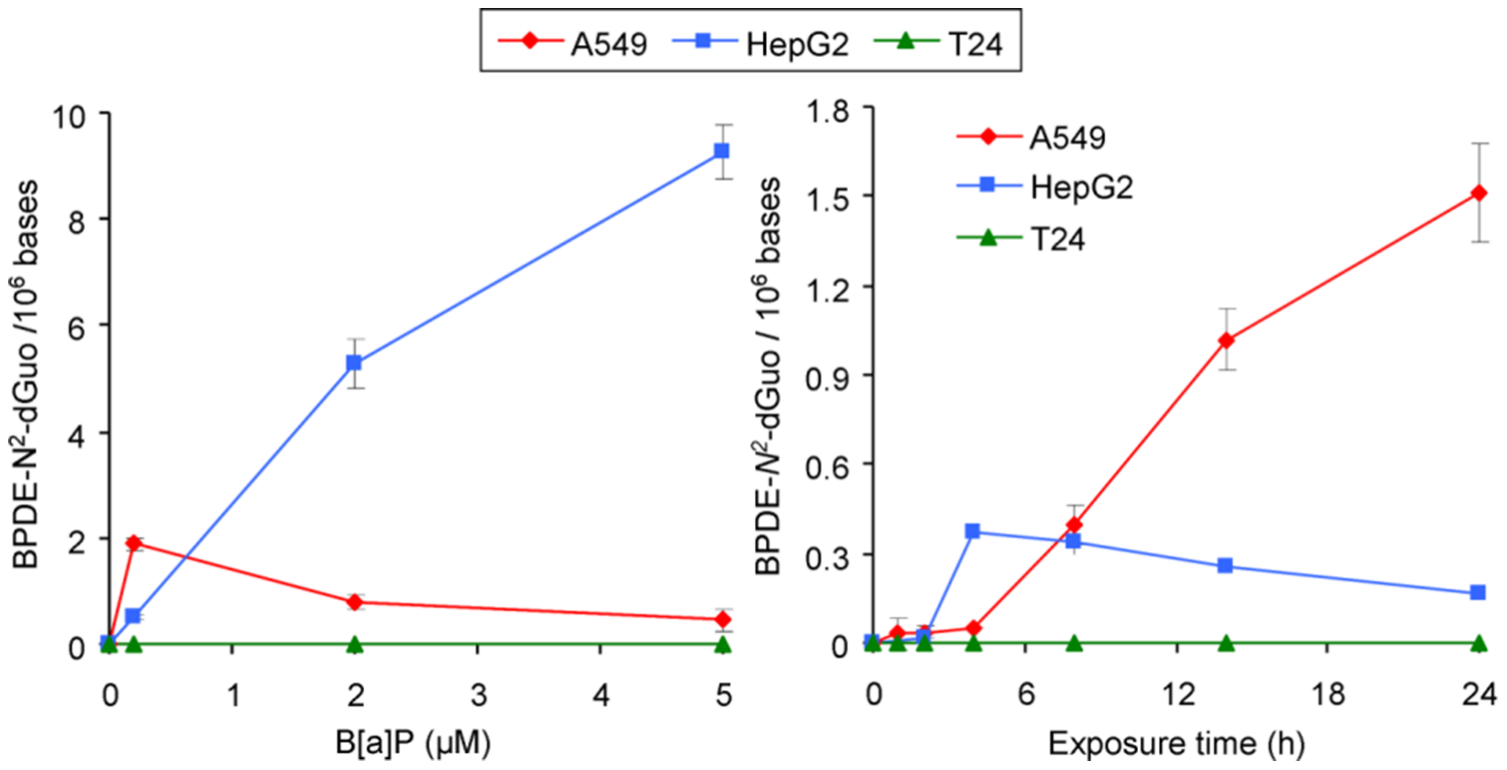
Dose-dependent (A) and time course formation (B) of BPDE-*N^2^*-dGuo adducts in HepG2, A549 and T24 cell lines exposed 14 h to increasing concentrations from 0.2 to 5 µM of B[a]P (A) or incubated with 0.2 µM from 30 minutes to 24 h (B).

The level of BPDE-*N^2^*-dGuo adduct was very different from one cellular model to the other. While adduct formation was dose dependent for HepG2 from 0.2 to 5 µM, the pattern observed in A549 was a bell-shaped response (Fig 1A). In this latter cell line, the level of DNA adducts reached a maximum at 0.2 µM and then continuously decreased as the B[a]P concentration increased to 5 µM. In order to confirm that 0.2 µM B[a]P was the actual concentration leading to the maximal level of adduct, an additional experiment was made with lower concentrations (Fig. S1). It should be emphasized that the amount of DNA adducts at 0.2 µM B[a]P was larger in A549 than in HepG2. Unlike the results obtained in these two cell lines, no BPDE-*N^2^*-dGuo was detected in T24 cells (Fig 1a), even when the B[a]P dose was increased to 10 and 20 µM (data not shown).

In order to determine the time dependence of the formation of BPDE-*N^2^*-dGuo adducts, cell lines were exposed from 30 minutes to 24 hours to 0.2 µM of B[a]P, the dose that induced the maximum amount of adducts in A549 cells (Fig 1B). The kinetic profiles were considerably different between the cellular models. A linear formation of adducts took place in A549 cells from 4 h to 24 h and slowed down after 24 h. Conversely, the amount of adducts observed in hepatocytes increased after a delay of 2 h, and then reached a plateau at 4 h until 24 h. Again, the adduct level was higher after 8 h in A549 than in HepG2. Another experiment confirmed the larger induction of BPDE-*N^2^*-dGuo adducts at 0.2 µM B[a]P than at higher concentrations over a 24 h period of time (Fig. S2). In T24 bladder cells, BPDE-*N^2^*-dGuo adduct could not be detected at any time even when the cells were exposed up to 20 µM for up to 24 h (data not shown).

### 2 Dose- and time-dependent formation of oxidative damage within cells exposed to B[a]P

**Figure 2:**
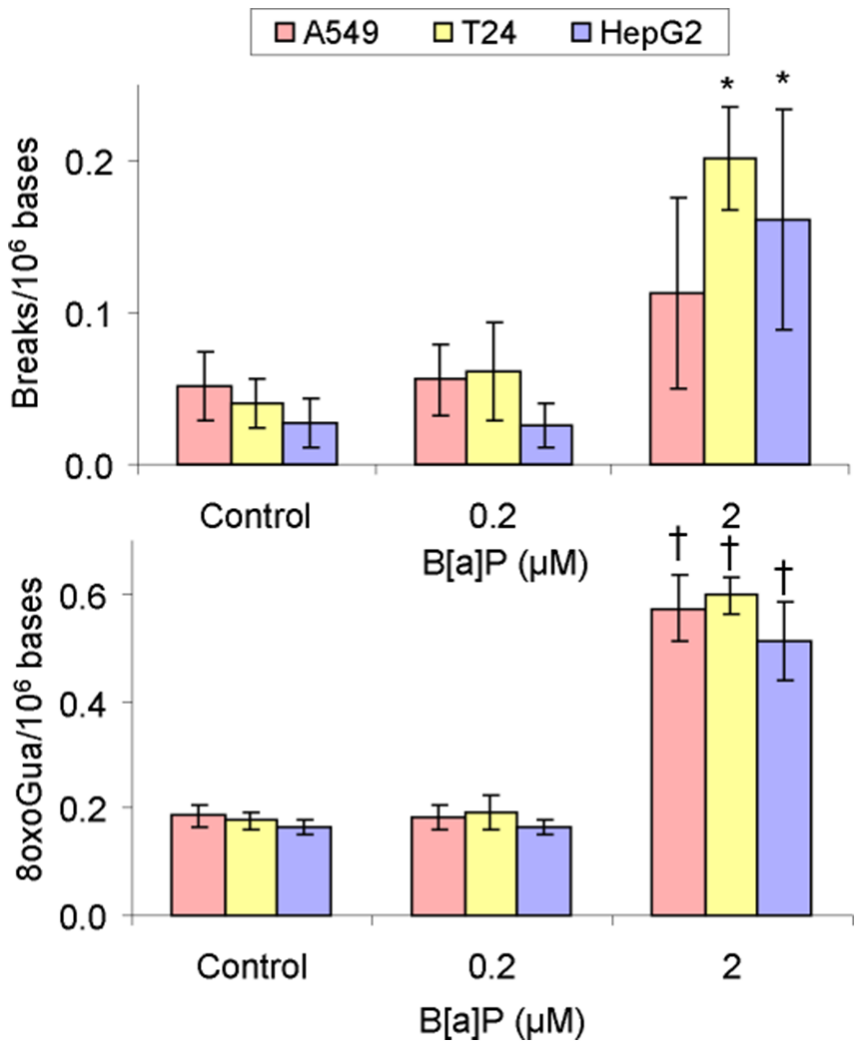
Level of oxidative DNA damage measures by a calibrated Comet assay in cells exposed to B[a]P for 14 h. Values for the strand breaks (A) were obtained from the standard version of the method while the level of 8-oxodGuo (B) was obtained with incubation of the slides with the Fpg repair enzyme. (Statistically significant (p<0.05) with respect to (*) DMSO control and (†) Fpg buffer control).

In a subsequent series of experiments, oxidative DNA damage resulting from exposure to B[a]P was measured in cells by the γ-radiation calibrated Comet assay in the presence or the absence of Fpg treatment. Similar results were obtained for the three cell lines investigated. In untreated cells, the background level of 8-oxodGuo was higher than that of strand breaks. Significant induction was observed at 2 µM, but not at 0.2 µM. The increase of damage at 2 µM was in the average of 0.1 break/10^6^ bases (Fig. 2A) and a larger induction of 0.4 8oxodGuo/10^6^ bases (Fig. 2B). It should be mentioned that we checked the induction of strand breaks by the Fpg buffer alone and observed a small increase of approximately 0.03 breaks/10^6^ bases (data not shown).

**Figure 3:**
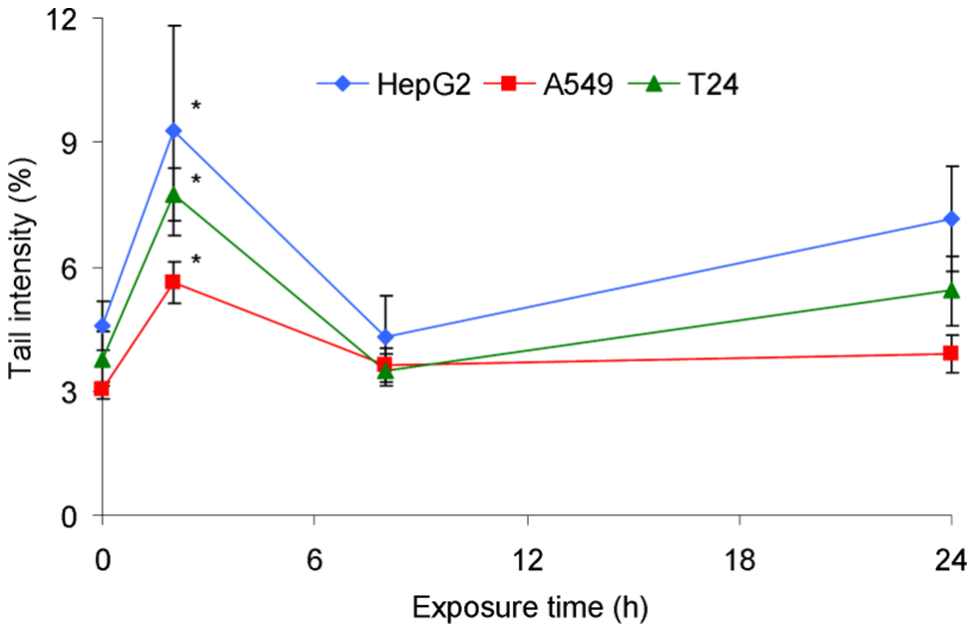
Time-dependent formation of DNA strand breaks measured by the Comet assay in HepG2, A549 and T24 cell lines incubated with 0.2 µM for a time period ranged between 30 min to 24 h. *: significantly different from t = 0 h (p<0.05).

The time- dependence was similar for the three cell lines exposed to 0.2 µM B[a]P from 30 minutes to 24 h (Fig. 3). A small but significant increase in the level of strand breaks relative to control was observed at 2 h. The basal level was restored at 6 h.

### 3 Metabolization of B[a]P in the three cell lines

In order to better understand the huge differences in adduct formation between the cell lines, the expression of cytochrome P450 oxygenases and epoxide hydrolase, the two enzymes involved in the formation of BPDE, was studied and the EROD activity quantified. Information was also gathered on GST, the main phase II enzyme involved in the BPDE detoxification pathway.

#### 3.1 Concentration-dependent expression of phase I enzymes in HepG2, A549 and T24

**Figure 4:**
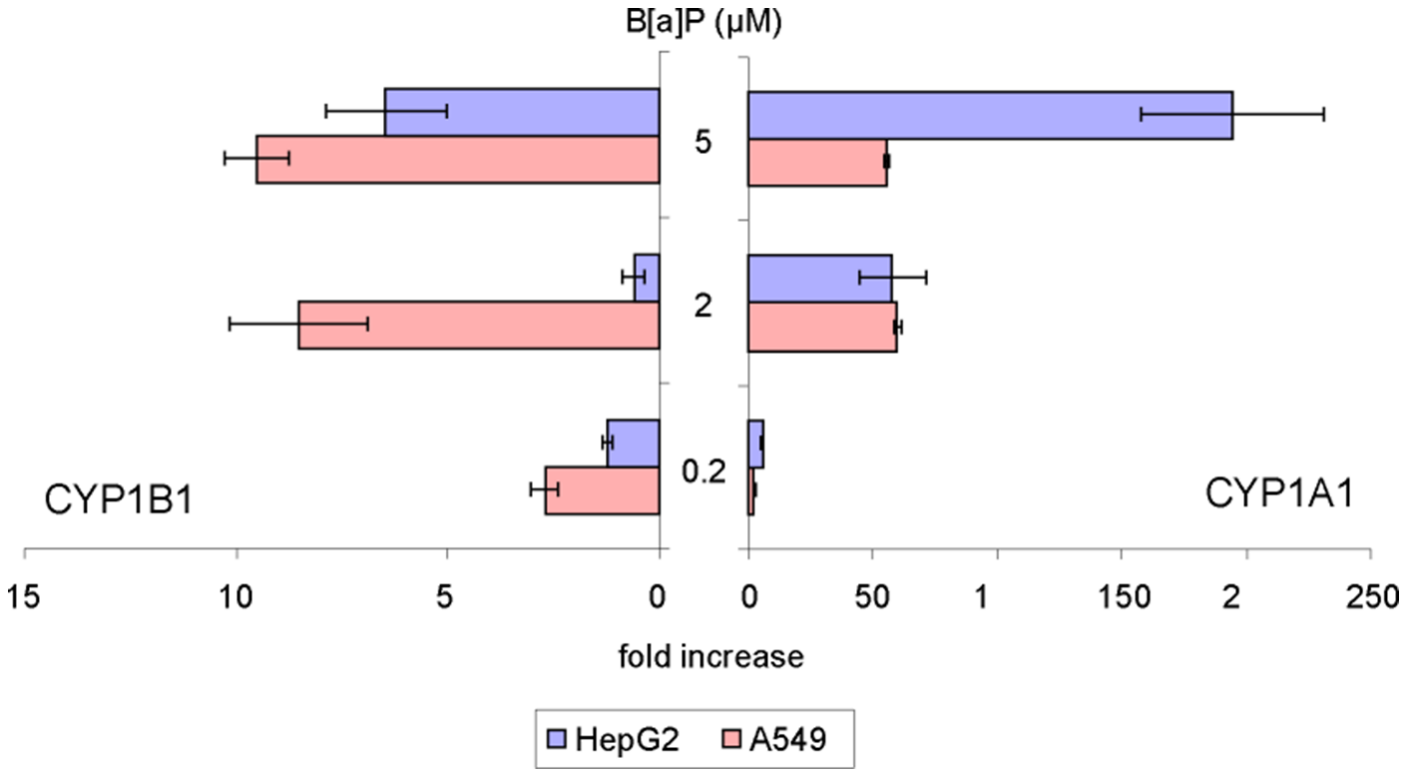
Expression of CYP1A1 (right) and CYP1B1 (left) genes in HepG2 and A549 cells exposed to increasing concentrations of B[a]P from 0.2 to 5 µM for 14 h. Differences with respect to DMSO control were found statistically significant for both genes in both cell lines at all concentrations (p<0.05) except CYP1B1 in HepG2 at 0.2 and 2 µM B[a]P.

**Figure 5:**
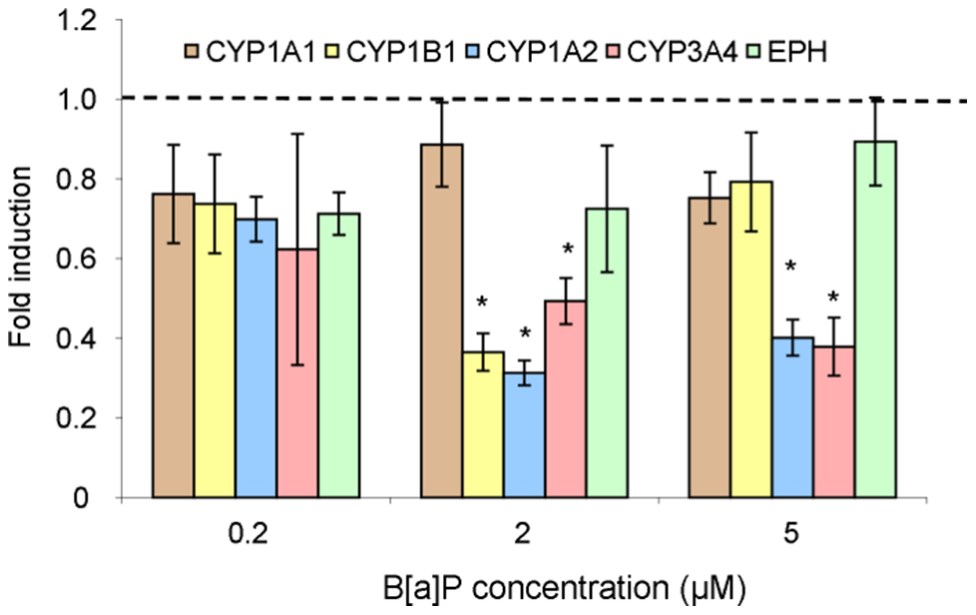
Modulation of gene expression in T24 cells exposed for 14 h to increasing concentrations of B[a]P from 0.2 to 5 µM. Statistically significant (p<0.05) with respect to (*) DMSO control).

Exposure to B[a]P markedly increased the level of CYP1A1 mRNA within HepG2 and A549 cells (Fig. 4). For both cell lines, a strong induction of CYP1A1 was observed at 2 µM with a 60 times higher value than in controls. While a plateau was reached within A549, induction in HepG2 was further enhanced to a 200 fold increase at 5 µM. CYP1B1 transcript level increased at 2 µM B[a]P and remained stable at 5 µM (induction fold of 9 relative to control) within A549 cells. In contrast, the over-expression of CYP1B1 was observed only for a treatment with 5 µM B[a]P in hepatocytes. Only limited modulation in the expression of other metabolization genes was observed (Table 1). In T24 cells, no significant over-expression of targeted genes was detected (Fig. 5), even repression was observed especially for CYP1B1, 1A2 and 3A4 at 2 µM and for 1A2 and 3A4 at 5 µM. Expression of EPH was found to remain unchanged in all cell lines at all B[a]P concentrations.

**Table 1:** Modulation of the expression of a series of phase I metabolization enzymes in HepG2 and A549 cells exposed for 14[a]P from 0.2 to 5 µM.

	HepG2			A549		
B[a]P (µM)	CYP1A2	CYP3A4	EPH	CYP1A2	CYP3A4	EPH
**0.2**	3.25±0.34 *	1.61±0.15	0.86±0.10	2.41±1.34	1.21±0.10	0.68±0.34
**2**	3.77±0.63 *	1.14±0.11	0.77±0.22	2.64±0.78	1.29±0.05	0.77±0.22
**5**	4.79±1.45 *	2.18±0.82	1.05±0.04	2.23±0.85	2.75±0.48 *	0.80±0.19

(Statistically significant (p<0.05) with respect to (*) DMSO control of the concerned cell line).

#### 3.2 Time-dependence of the modulation of the expression of phase I enzymes in A549 and HepG2 cells

**Figure 6:**
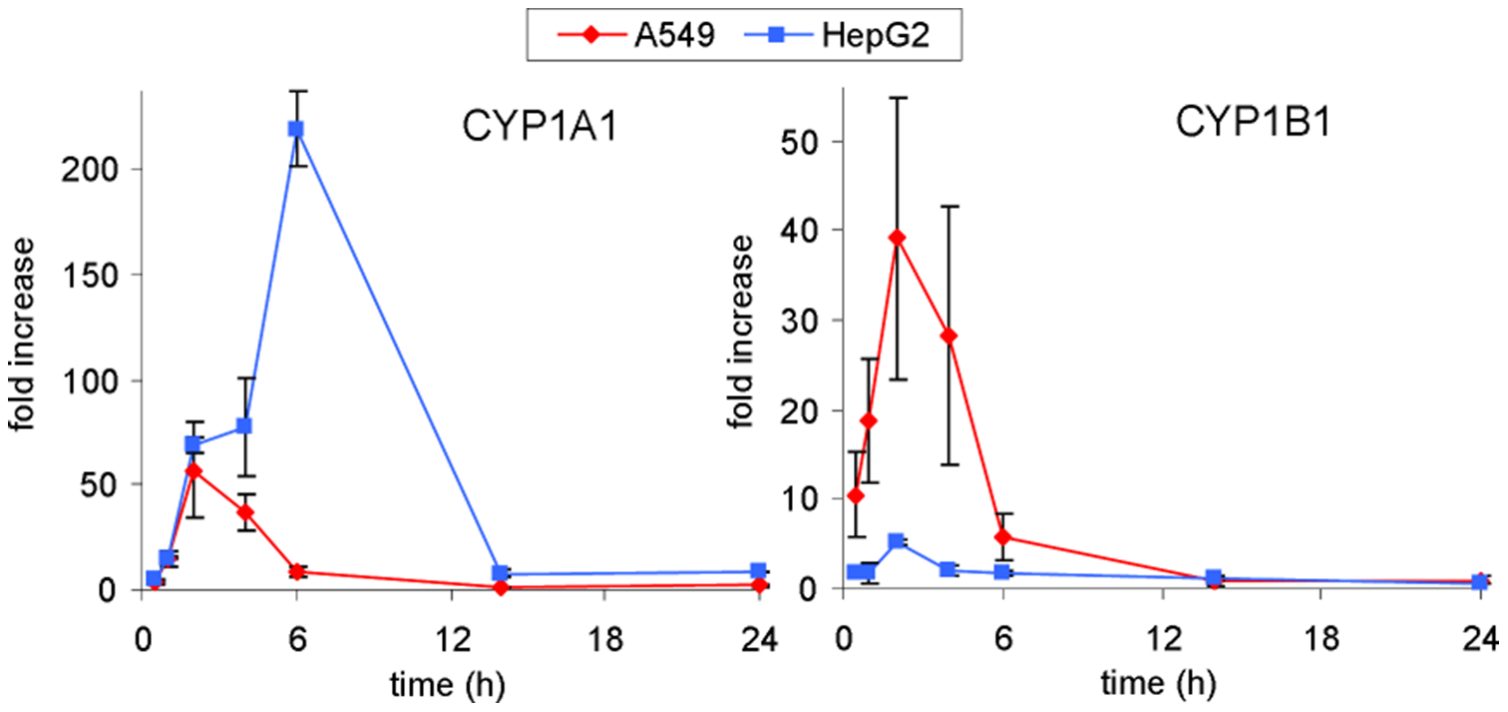
Induction of CYP1A1 and 1B1 in A549 and HepG2 cells exposed to 0.2 µM B[a]P. Data were found significantly different (p<0.05) with respect to DMSO control for both cell lines between 30 min and 6 h.

Time-dependent expression of metabolism enzymes was investigated for a period ranging from 30 min to 24 h. The concentration of B[a]P was 0.2 µM because it corresponded to the maximum of DNA adduct formation in A549. T24 cells were not included in this study because of the absence in detected adducts and the lack of variation in gene expression in the dose-dependence study at 14 h reported above. In HepG2, the level of CYP1A1 mRNA strongly increased with exposure time until 6 h (219±18 induction fold) and then drastically decreased after 14 h of treatment (Fig. 6). A much more modest over-expression of CYP1B1 and CYP1A2 (expression ratio of 5 and 8) was observed with a maximum at 2 h and 6 h of treatment respectively. No overexpression of CYP3A4 and EPH was detected. In A549 maximal induction for CYP1A1 and 1B1 was reached at 2 h with levels 4-times lower and 8-times higher, respectively, than those observed in HepG2 (Fig. 6). The induction of both genes was 5-times lower at 6 h than at 2 h. No significant modulation was detected in A549 for the other transcripts (Table 2).

**Table 2:** Modulation of phase I metabolism enzymes in HepG2 and A549 cells following exposure to 0.2 µM B[a]P.

	HepG2			A549		
Exposure time (h)	CYP1A2	CYP3A4	EPH	CYP1A2	CYP3A4	EPH
**0.5**	0.66±0.28	1.94±0.12 *	0.82±0.23	2.41±1.34	2.10±0.86	0.67±0.28
**1**	1.52±0.93	0.96±0.55	0.60±0.41	1.16±0.58	1.18±0.39	0.35±0.12
**2**	1.70±0.30 *	1.56±0.28	0.80±0.21	1.68±0.85	1.01±0.32	0.75±0.19
**4**	4.47±0.31 *	1.59±0.17	0.80±0.11	1.40±0.67	1.06±0.37	0.71±0.21
**6**	7.39±0.73 *	1.43±0.21	0.93±0.14	1.34±0.78	1.37±0.41	0.90±0.13
**14**	4.76±0.94 *	1.42±0.22	0.91±0.09	0.67±0.46	1.38±0.51	0.72±0.22
**24**	4.88±0.49 *	2.12±0.07 *	0.80±0.14	1.61±0.85	1.98±0.89	0.75±0.31

* : significantly different from DMSO control of concerned cell line (p<0.05).

#### 3.3 EROD activity

Incubation times were selected on the basis of the work conducted by Billet *et al.* [26] who showed that the maximum EROD activity was recorded at 24 h in HepG2 and 48 h in A549. We found a similar EROD activity in HepG2 and A549 in response to 0.2 µM B[a]P (Table 3). In contrast a large difference was observed at 2 and 5 µM with an order of magnitude difference. In addition, the dose response was different, with a trend to a plateau at 2 and 5 µM for lung cells and a roughly linear dose-response for hepatocytes.

**Table 3:** Increase in EROD activity^a^ measured in HepG2 and A549 cells exposed to B[a]P from 0.2 to 5 µM.

B[a]P (µM)	0	0.2	2	5
**A549**	0.00±0.08	0.28±0.08	0.46±0.11	0.59±0.17
**HepG2**	0.00±0.05	0.30±0.11	3.49±0.61	7.25±0.45

Exposure time was 24 h for HepG2 and 48 h for A549.

a: expressed in pmol of product/min/mg of protein. The average background measured in untreated cells value was subtracted.

#### 3.4 Expression and activity of GSTs

**Figure 7:**
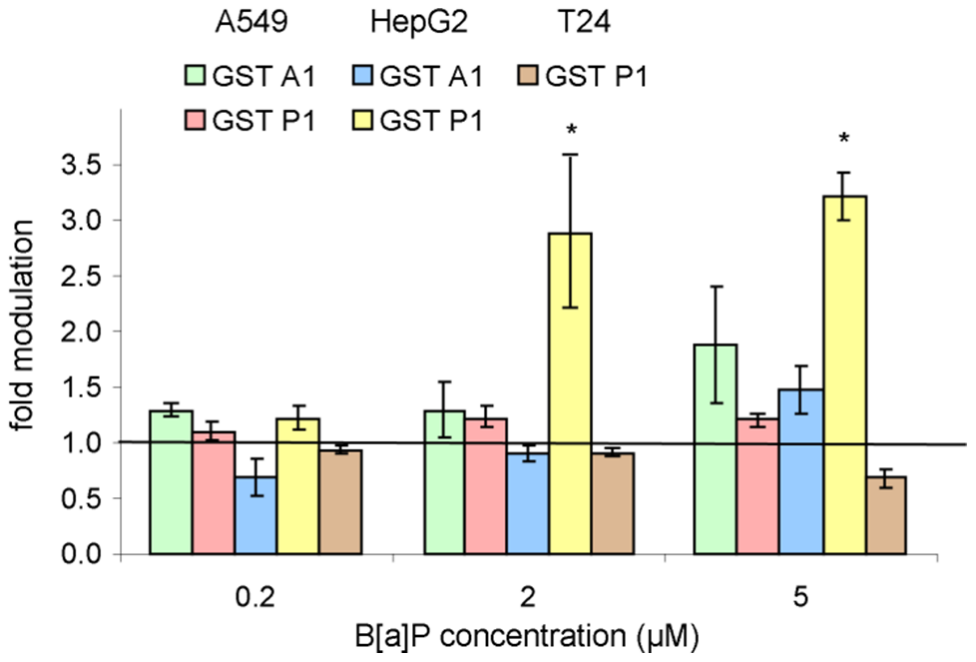
Modulation of the expression of a series of GST genes in HepG2, A549 and T24 cells exposed to increasing concentrations of B[a]P from 0.2 to 5 µM. *: significantly different from DMSO control (p<0.05). Expression of GSTA1 was not detected in T24.

Limited modulation of the expression of the genes coding for GSTs was observed in the three cell lines with only a 3-fold induction of GSTP1 in HepG2 cells (Fig. 7). None of the investigated GSTs were modulated in T24 cells. This result was confirmed in the study of the time-dependence where only a small induction of GSTP1 was observed in cells at 2 h (Table S2).

In agreement with these data, the GST activity remained constant for all concentrations (Table 4) and incubation times (figure S3). In addition, similar basal levels of activity were determined for the three cell types.

**Table 4:** GST activity^a^ in HepG2 and A549 cells exposed to B[a]P from 0.2 to 5 µM.

		HepG2	A549
Dose B[a]P (µM)	0	0,24±0,02	0,30±0,03
	0,2	0,19±0,03	0,26±0,02
	2	0,18±0,02	0,28±0,03
	5	0,22±0,03	0,28±0,02

a: results are expressed in µmol of product/min/mg of proteins.

#### 3.5 Expression of AKRC1 and MRP4 genes

**Figure 8:**
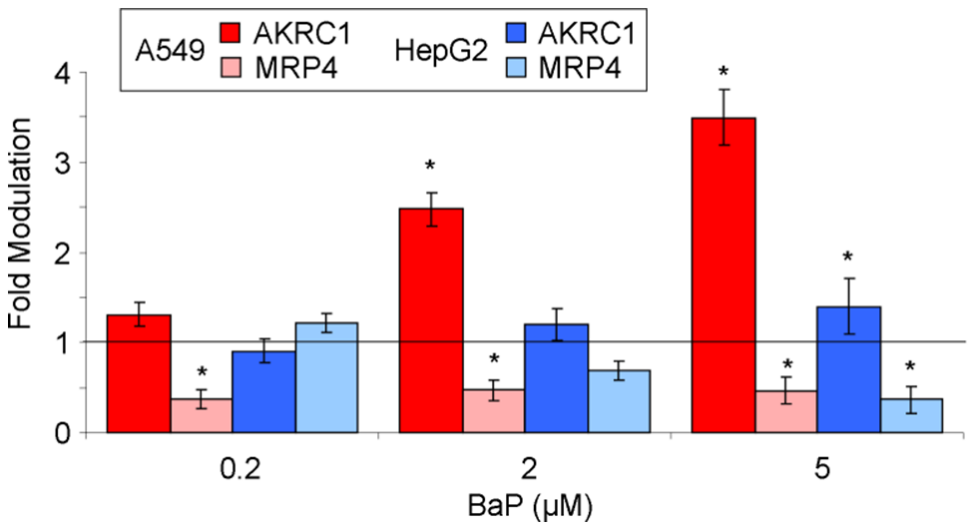
Modulation of the expression of the gene coding for AKRC1 and MRP4 in A549 and HepG2 cells exposed for 14 h to increasing concentrations of B[a]P from 0.2 to 5 µM. Results were expressed with respect to untreated control.

Last, we quantified the expression of the genes coding for AKRC1 and MRP4. These proteins are involved in the detoxification of the B[a]P mono-epoxide by conjugation to GSH and export out of the cell, respectively. At the basal level, the mRNA of both proteins were found to be more than 10 times more frequent in A549 than in HepG2. This difference was increased for AKRC1 upon exposure to B[a]P since significant over expression compared to untreated control was observed at 2 and 5 µM in A549 but not in HepG2 (Fig. 8). The expression of MRP4 was found to be lowered in both cell lines by addition of B[a]P but not to an extent which could compensate for the initial larger expression in A549.

## Discussion

Because animal studies are expensive, time-consuming and of limited use because of regulatory issues, development of reliable cellular tests is an obviously interesting and widely developed alternative. Yet, the question is raised of the relevance of the cellular models. For the study of PAH genotoxicity, hepatocytes have been widely used because they express a large part of the metabolic activities required for phase I and II metabolization [27], [28], [29], [30]. PAHs do not induce liver cancers whereas lungs, and to a lesser extent bladder and skin are the main target organs. We therefore designed this study to compare the DNA damage induced by B[a]P in pulmonary and bladder cell lines with those in widely studied HepG2 hepatocytes. All experiments were performed at low concentrations of B[a]P in order to be close to biologically relevant exposure conditions and to limit the impact on cellular viability. Indeed, at least 70% of cells are viable in our experiments (data not shown). Concentration is also an important parameter to consider when comparing cell lines, as shown for example by the difference in the dose dependence of mutation profile in cells exposed to PAHs diol epoxides [31], [32].

The A549 cell line was chosen as *in vitro* pulmonary model. This cell line is a type II pneumocyte which is extensively used in toxicology [26], [33], [34], [35]. Genotoxicity was investigated both in terms of BPDE adducts and oxidative DNA damage. In a concentration-effect study, we obtained a bell-shaped curve with a maximal induction of DNA adducts at 0.2 µM. This behaviour contrasts with the formation of adducts proportional to the B[a]P concentration observed in HepG2, in agreement with our previous observations [16]. These contrasting results could not be explained by a bias related to the time of analysis, as shown by studies of the time dependence of the formation of BPDE-*N^2^*-dGuo in both cell lines. A concentration dependence with a maximal adduct formation at low concentration has been observed in other lung models. The same results were reported in HEL (Human diploid lung fibroblasts) and BEAS-2B (Bronchial epithelial cells) exposed to increasing doses of B[a]P to 10 µM for 24 h or 48 h [18],[36]. The maxima were observed at 1 µM and 2.5 µM respectively. Interestingly, these authors also observed a higher level of BPDE adducts in lungs cells than in HepG2 hepatocytes upon exposure to 0.2 and 1 µM B[a]P.

A possible explanation to the difference in DNA adduct formation in A549 and HepG2 could be a drastic difference in B[a]P intake between the two cell lines. It was however shown that A549 cells are only 2 fold less efficient than HepG2 cells in that respect [37]. We also observed that the levels of adducts in the three investigated cell lines exposed to pure BPDE were of the same order of magnitude (data not shown), suggesting similar uptakes of polycyclic aromatic molecules. It is thus most likely that the differences in the patterns of induction of BPDE adducts reflect the differences in time- and concentration-dependent metabolic responses of A549 and HepG2 to B[a]P treatment. We thus investigated B[a]P metabolism in terms of gene expression coding for metabolic enzymes and phase I (EROD) and II (GST) enzymatic activities.

While the CYP1A1 mRNA expression reached a plateau at 2 µM B[a]P in A549 cells treated for 14 h, it was proportional to the B[a]P concentration in HepG2. In terms of kinetics, we observed that the CYP1A1 expression was less intense at its maximum and less persistent in A549 than in HepG2 upon exposure to 0.2 µM. All these gene expression data were in accordance with EROD activity. These differences are in agreement with published works showing that the distribution of radioactive metabolites was similar in the two cell lines but that the overall amount was larger in HepG2 [37]. These cellular observations reflect what happens *in vivo*, with liver being the favoured location for metabolization. This tissue specific metabolism was also observed in rat lung and liver slices. Maximal induction of metabolization was reached at 1 µM B[a]P in lung slices whereas it required 10 µM for liver slices suggesting better metabolizing capacities in the latter organ [38]. This result correlated with an induction of CYP1A1 mRNA proportional to the B[a]P dose in liver slices and with the observation of a plateau for lung slices at 1 µM. Furthermore, EROD activity was 10 times weaker in lung slices than in liver slices. The observed saturation of CYP1A1 mRNA induction and EROD activity in A549 may be linked to AhR expression, a key player in B[a]P metabolization by activating multiple enzyme expressions like CYP1A1, 1B1, GST and efflux proteins [12], [39], [40], [41]. AhR is detected in a majority of tissues but at different levels [42], [43]. Iwanari *et al* [44] showed a weaker expression of AhR in A549 than HepG2. The same observation was made when comparing slices of rat liver and lungs [45], which is in line with the idea that hepatocyte cell lines are more metabolically active than A549 towards B[a]P. However, it should be stressed that the differences in CYP1A1 expression and EROD between HepG2 and A549 is lower at low B[a]P concentration, in conformation with the larger amount of BPDE adducts observed in A549 at 0.2 µM B[a]P.

Furthermore, induction of genes other than CYP1A1 was different in the two cell lines. The most significant differences were observed for CYP1B1 the induction of which by 0.2 µM of B[a]P was faster and 8 times higher in A549 than in HepG2. This is in agreement with the different pattern of altered genes observed between human hepatic and pulmonary cells exposed to 1 µM B[a]P [17]. The same observation of a larger expression of CYP1B1 in lung compared to liver has been reported in rat samples [46]. CYP1B1 has been shown to be more efficient than CYP1A1 at converting B[a]P into its 7,8-diol derivative [47] and to exhibit a larger mRNA half-life than CYP1A1 [48]. CYP1B1 is thus likely to play a significant role in the formation of BPDE DNA adducts in A549. Another CYP450, CYP1A2, was also differentially expressed in the two cell lines in response to B[a]P, with a significant induction in HepG2 although in a 20-fold lower extent than CYP1A1. Incubation with B[a]P had only minor impact on other phase I enzymes, such as other CYP and epoxide hydrolase.

In contrast to CYP450, no difference was found in the expression and activity of GST, which is one of the most efficient protective enzymes against PAHs epoxides. These cytosolic enzymes are widely distributed in various tissues, with especially high levels in the liver [49], [50]. Neither the expression of GST genes nor the global GST activity were greatly impacted by B[a]P treatment of A549 and HepG2. This absence of modulation was reported in other studies where phase I was found to be far more inducible than phase II [49], [51], [52]. Indeed, no modulation in GST activity was observed in rat lung and liver slices exposed to a B[a]P concentration as high as 50 µM during 24 h using CDNB. Yet, the resulting GST activity was around 10 times higher in liver than in lungs slices [53]. In contrast, we found similar basal level activity in the A549 and HepG2 cell lines.

The bulk of these data partly explains the differences in dose-dependent formation of BPDE adducts in A549 and HepG2. A much higher induction of CYP1A1 is observed in hepatocytes, which likely explains the large level of adducts at 2 and 5 µM. The lower induction of CYP1A1 in A549 is partly compensated by the larger expression in CYP1B1. This may explain why the ratio between the EROD activities in the two cell lines is closer to 1 at 0.2 µM than at 2 or 5 µM. These observations may also explain why a significant formation of adducts is observed at low concentration in A549. However, in the absence of a role of phase II enzymes, it is difficult to explain the bell-shaped curve of the concentration dependence of adduct formation in A549 cells, especially the decreased level of adducts at high concentration. One explanation could be that AKRC1 is massively expressed in pulmonary cells and in A549. This enzyme converts 7,8-diol-7,8-dihydro-benzo[a]pyrene into the corresponding *o*-quinone [33], [54], [55]. Similarly, the gene for MRP4, a protein that eliminates B[a]P diol, is more expressed at the basal level in A549 than in HepG2. This trend is still observed after incubation with B[a]P although partial down regulation of the expression takes place in both cell lines. It can thus be considered that the pool of free BPDE is limited in A549 at high concentration of B[a]P as the result of the larger expression of these two proteins eliminating the first intermediate in the diol-epoxide pathway.

In addition to lung cancer, PAHs are also suspected to be risk factors for bladder cancer in several occupations [3], [6], [56] but the underlying mechanism is still poorly understood. Bladder exposure to toxic substances obviously involves metabolites produced in other tissues but urothelium should not be considered as a passive target organ for carcinogenic intermediates. Indeed, the expression of several detoxification enzymes especially CYP1A1 or B1 has been reported and this tissue is capable of actively metabolizing chemical substances such as PAHs [57]. In order to clarify the PAHs mode of action in bladder carcinogenicity, we found it of interest to compare the effects of B[a]P in a bladder cell line with those in hepatocytes and lung cells. T24 cells, originating from a highly malignant grade III human urinary bladder carcinoma, are one of the few available bladder models and to our knowledge these cells have not previously been used for the investigation of B[a]P-induced genotoxicity. Exposure of T24 cells to B[a]P did not result in the formation of BPDE-DNA adducts within a broad range of doses and exposure times. In addition no induction of phase I metabolization enzymes was observed. The lack of adducts could not be explained by a more efficient phase II since neither gene expression nor activity was found to increase for GST after exposure to B[a]P. In addition, the basal GST activity was similar to that of HepG2 and A549. A last possibility to explain the absence of BPDE adducts is that the metabolic mechanisms triggered by B[a]P are different from those in liver and lung cells. If so, they are likely to be poorly genotoxic pathways because no unstable N7 adducts like those produced by the radical cation were detected in the Comet assay. The bulk of these negative results made us wonder whether B[a]P actually entered the cells. We thus used BPDE as a model compound and found that the level of adducts in T24 was in the same range as those in BPDE-treated HepG2 and A549 (data not shown), suggesting a similar uptake of PAH by the three cell lines. Our data thus clearly show that B[a]P is incorporated but induces a weak genotoxicity in T24 cells.

Yet, other bladder models have shown that PAHs could be converted into DNA damaging metabolites in this organ. Studies on Primary porcine Urinary Bladder Epithelial Cells (PUBEC) showed that bladder cells are able to concentrate B[a]P which is absorbed even at concentrations as low as 0.1 µM. Wolf *et al* [58] also reported a 200 fold increase of CYP1A1 mRNA expression after an exposure of 5 µM during 5 h. It should be stressed that a strong inter-individual and even inter-pool variability was observed for B[a]P uptake [59] and metabolism especially in CYP1A1 induction. For example, Plöttner *et al* [60] reported a 162 to 982-fold variation in the induction of CYP1A1 for a 5 µM exposure during 24 h for 15 cultures from different donors. In further analyses, these authors also established that PUBEC consisted of two distinct populations where CYP1A1 was inducible/or not and with large inter-individual differences in the proportion of responsive cells [61]. Similar variability has been observed also in humans, with different genetic susceptibilities in particular for CYP1A1 expression in normal urothelium [57]. Studies of human bladder explants exposed to B[a]P also showed the formation of metabolites such as BPDE and of DNA adducts yet with a considerable variability. The observation of formation of BPDE adducts in explants [62], [63] and not in T24 cells strongly suggest that a subpopulation of cells in bladder possesses inducible CYP450.

Beside the formation of BDPE adducts extensively discussed above, B[a]P has been proposed to be genotoxic through the induction of oxidative stress. Indeed, reactive oxygen species are produced by the activity of cytochrome P450 enzymes as well as by the redox cycling of metabolites. We decided to study the contribution of this oxidative pathway because *o*-quinones, metabolites known to induce oxidative DNA damage [64], were expected to be found in significant amounts in A549 through the action of AKRC1 and because metabolization was very active in HepG2. In addition, we wanted to find out if the lack of a metabolic pathway leading to production of BPDE in T24 was not compensated by an alternative mechanism resulting in oxidative DNA lesions. As previously reported for HepG2 [16], no significant formation of strand breaks and oxidized purines was observed at low concentration (0.2 µM) of B[a]P in the three cell lines. Under the same conditions, the level of BPDE-*N^2^*-dGuo was in the range of 1 per million normal bases in A549 and HepG2. At 2 µM B[a]P, significant and identical levels of oxidative lesions were determined in the three cells types. As previously observed, the ratio between adducts and induced strand breaks was very high (value: 41) in HepG2. A ratio of 15 was found between the amounts of BPDE-*N^2^*-dGuo and induced 8-oxodGuo. The corresponding data for the ratio between the level of adduct and induced oxidative lesion in A549 was 18 for strand breaks and 3 for 8-oxodGuo. It should however be reminded that this relatively high concentration is beyond the maximum of induction of BPDE-*N^2^*-dGuo in A549. In summary, oxidative DNA damage represents only a minor contribution to the genotoxicity of B[a]P in the three studied cell lines at low biologically relevant concentrations. Oxidative lesions are induced in larger amounts at higher concentrations. In A549 and HepG2, the frequency of oxidative lesions remains much lower than the frequency of BPDE adducts. In line with this conclusion, exposure of human bronchoalveolar H358 cells to the *o*-quinone benzo[a]pyrene-7,8-dione was shown to lead to the formation of only 0.4 8-oxodGuo per 10^6^ normal bases at 20 µM [65]. This concentration is 2 orders of magnitude higher than that required to induce a 5 time larger amount of BPDE adducts in A549 cells. The limited oxidative stress associated with B[a]P treatment in cultured cells may explain the poor correlation between exposure of workers to PAH-rich atmospheres and markers of DNA oxidation [66], [67].

## Conclusion

The present study emphasizes the drastic differences in the response of different human cell types to B[a]P exposure. In particular, our results show that concentration is a key parameter and thus that erroneous conclusions can be made. For instance, A549 cells, like lungs and other pulmonary models are metabolically active and undergo a formation of significant amounts of DNA adducts more efficiently at a low and biologically relevant concentration than at higher concentration. In that respect A549 appear as a good model for lung genotoxicity of PAH. T24 bladder cells, another target organ for PAHs, are much less metabolically active and no adducts were found after exposure to B[a]P likely because these cells do not metabolize these compounds. However, T24 represent only a subpopulation of bladder cells and cannot be considered as a reliable model for this organ. An important conclusion of the present work is the minor implication of oxidative stress as a genotoxic pathway of B[a]P. Comet assay does not appear to be a good test for PAH genotoxicity studies because it is only suitable at concentrations higher than 1 µM and furthermore detects only a minor part of the DNA damage. Obviously, these conclusions do not apply to all compounds but should help us to realize the difficulty to define a universal and global genotoxicity assay.

## Supporting Information

Table S1 **Primers used for qPCR experiments.**
(PDF)

Table S2 **Time-dependent modulation of GST expression in A549 and HepG2 cells exposed to 0.2** **μM B[a]P.**
(PDF)

Figure S1 (TIF)

Figure S2 (TIF)

Figure S3 (TIF)
