# Supplementary material for: The Extreme Variety of Genotoxic Response to Benzo[a]pyrene in Three Different Human Cell Lines from Three Different Organs
Source: PLoS One. 2013 Nov 8;8(11):e78356. doi: 10.1371/journal.pone.0078356 (PMC3832631; doi:10.1371/journal.pone.0078356)
Supplement: Table S1 — Primers used for qPCR experiments. (PDF) [file pone.0078356.s001.pdf]

Table S1: primers used for qPCR experiments

Gene	Forward primer	Reverse primer	Product Size (pb)
GAPDH	GAGTCAACGGATTTGGTCGT	TTGATTTTGGAGGGATCTCG	238
S18	AACGTCTGCCCTATCAACTTT	TGGATGTGGTAGCCGTTTTCT	117
CYP1A1	CTTGGACCTCTTTGGAGCTG	CGAAGGAAGAGTGTCGGAAG	212
CYP1B1	GACCCCCAGTCTCAATCTCA	CTCCCACTCGAGTCTCTTGG	152
CYP1A2	GGACAGCACTTCCCTGAGAG	GTCCCGGACACTGTTCTTGT	231
CYP3A4	GCCTACAGCATGGATGTGAT	CGAGGCGACTTTCTTTCATC	250
EPH	CAGAGGCATCCTCCAAGAAG	GCAGGAGGGTCAGGGTAGAG	219
GSTA1	CGGTGACAGCGTTTAACAAA	GCACTTGCTGGAACATCAAA	247
GSTP1	ACCTCCGCTGCAAATACATC	GGCTAGGACCTCATGGATCA	206
AKRC1	AGATTGCAGATGGCAGTGTG	TCACCTTCCTCACCTGGCTTT	183
MRP4	GTTGCAAGGGTTCTGGGATA	GCTGTGTTCAAAGCCACAGA	225
