# Supplementary material for: The Extreme Variety of Genotoxic Response to Benzo[a]pyrene in Three Different Human Cell Lines from Three Different Organs
Source: PLoS One. 2013 Nov 8;8(11):e78356. doi: 10.1371/journal.pone.0078356 (PMC3832631; doi:10.1371/journal.pone.0078356)
Supplement: Table S2 — Time-dependent modulation of GST expression in A549 and HepG2 cells exposed to 0.2 μM B[a]P. (PDF) [file pone.0078356.s002.pdf]

Table S2: time-dependent modulation of GST expression in A549 and HepG2 cells exposed to 0.2 µM B[a]P.

	HepG2		A549	
Exposure time (h)	GSTA1	GSTP1	GSTA1	GSTP1
0.5	1.21 ± 0.07	0.58 ± 0.13	1.06 ± 0.45	1.09 ± 0.50
1	1.27 ± 0.01	0.68 ± 0.54	0.86 ± 0.22	1.05 ± 0.44
2	1.28 ± 0.02	2.99 ± 0.95 *	0.75 ± 0.25	1.21 ± 0.44
4	1.41 ± 0.12	0.92 ± 0.07	0.98 ± 0.32	0.33 ± 0.16 *
6	1.27 ± 0.01	2.30 ± 0.65	1.21 ± 0.19	0.54 ± 0.33
14	1.26 ± 0.14	0.66 ± 0.34	0.70 ± 0.23	0.60 ± 0.26
24	1.38 ± 0.02	0.55 ± 0.06	1.32 ± 0.52	0.66 ± 0.31
