# Supplementary figures and images for: The Extreme Variety of Genotoxic Response to Benzo[a]pyrene in Three Different Human Cell Lines from Three Different Organs
Source: PLoS One. 2013 Nov 8;8(11):e78356. doi: 10.1371/journal.pone.0078356 (PMC3832631; doi:10.1371/journal.pone.0078356)

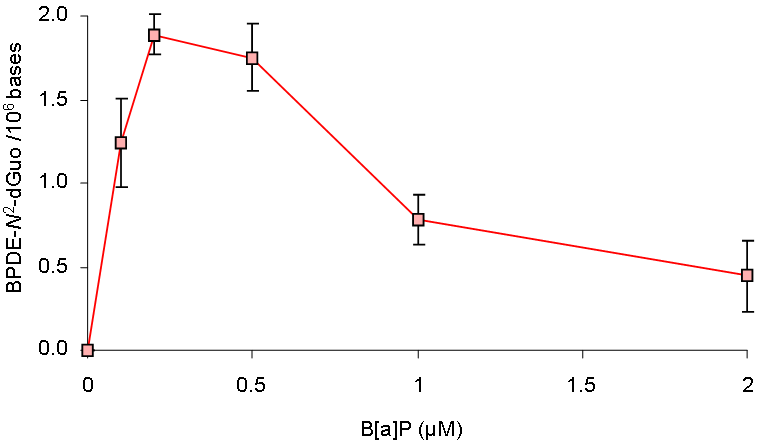

Supplement: Figure S1 — (TIF) [file pone.0078356.s003.tif]

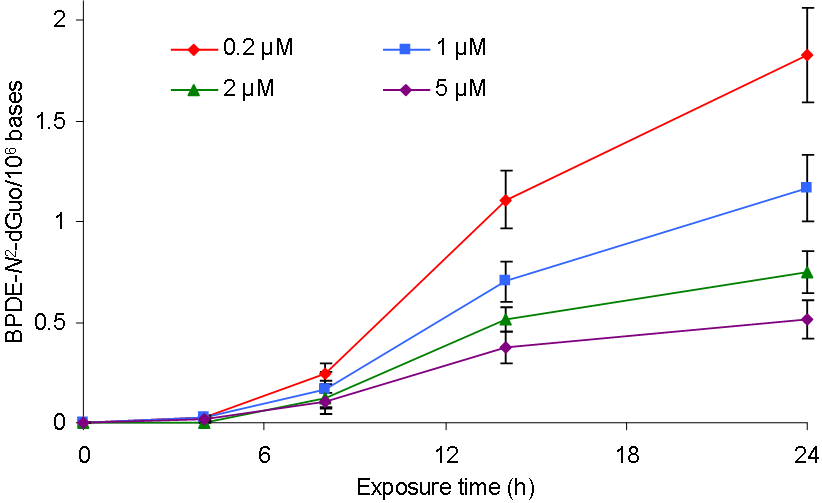

Supplement: Figure S2 — (TIF) [file pone.0078356.s004.tif]

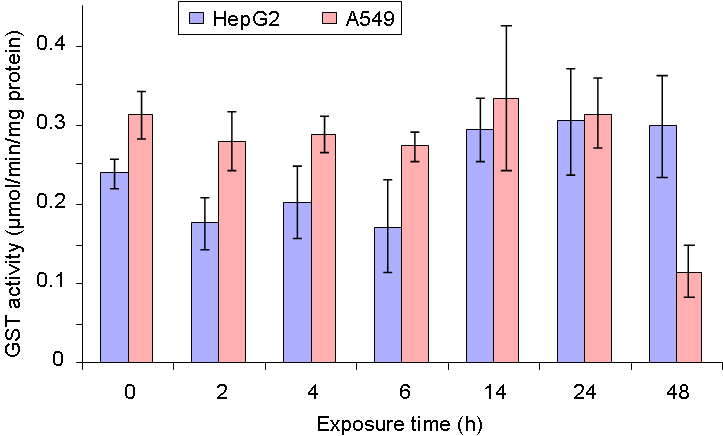

Supplement: Figure S3 — (TIF) [file pone.0078356.s005.tif]
